# Only distance matters – non-choosy females in a poison frog population

**DOI:** 10.1186/1742-9994-10-29

**Published:** 2013-05-20

**Authors:** Ivonne Meuche, Oscar Brusa, K Eduard Linsenmair, Alexander Keller, Heike Pröhl

**Affiliations:** 1 Institute of Zoology, University of Veterinary Medicine, Bünteweg 17d, 30559, Hannover, Germany; 2 Department of Animal Ecology and Tropical Biology, Biozentrum, University of Würzburg, 97074, Würzburg, Germany; 3 DNA Analytics Core Facility and Department of Animal Ecology and Tropical Biology, Biozentrum, University of Würzburg, 97074, Würzburg, Germany

## Abstract

**Background:**

Females have often been shown to exhibit preferences for certain male traits. However, little is known about behavioural rules females use when searching for mates in their natural habitat. We investigated mate sampling tactics and related costs in the territorial strawberry poison frog (*Oophaga pumilio*) possessing a lek-like mating system, where both sequential and simultaneous sampling might occur. We continuously monitored the sampling pattern and behaviour of females during the complete period between two successive matings.

**Results:**

We found no evidence that females compared males by visiting them. Instead females mated with the closest calling male irrespective of his acoustic and physical traits, and territory size. Playback experiments in the natural home ranges of receptive females revealed that tested females preferred the nearest speaker and did not discriminate between low and high call rates or dominant frequencies.

**Conclusions:**

Our results suggest that females of *O*. *pumilio* prefer the closest calling male in the studied population. We hypothesize that the sampling tactic in this population is affected by 1) a strongly female biased sex ratio and 2) a low variance in traits of available males due to strong male-male competition, preventing low quality males from defending a territory and mating.

## Background

The function of extravagant male characters and females preferences for these traits has been a major focus of research in behavioural and evolutionary biology [1]. In recent years, particular attention has been concentrated on determining the origin and adaptiveness of female mating preferences. It has been demonstrated that females can gain direct [2] and indirect benefits [3-6] from choosing among males and from their preferences for certain male traits.

Despite a steady increase in empirical and theoretical investigations about sexually selected traits [7], relatively few studies have been devoted to information gathering by females about male traits and decision making based on that information [8,9]. Detailed knowledge about female mate sampling behaviour is important because it directly affects the evolutionary trajectory of male characteristics [10-12]. Such knowledge is also important to understand how selection acts on female mating preferences, since the expression of mating preferences depends on the mate sampling tactic. Several theoretical models as to how females sample potential mates have been proposed [10,11,13-18]. The simplest and least costly female mate sampling tactic (in terms of time and energy expenditure) is to mate with the first male encountered [10]. This random mating implies that accepted males can be of any quality [19]. Alternatively, females may use a search tactic, in which they compare a number of males and then choose the male with the highest quality within their sample [20]. Under this scenario, females may compare only the last encountered males [21,22], a certain number of males ["best-of-N" tactic: 10] [11,17] or sample males for a certain amount of time [13] before returning to the highest quality male. The quality of accepted males should be higher for the respective female than the quality of rejected males [17]. Females may also use a threshold tactic whereby the first male that exceeds an established threshold criterion is accepted [10,11,22]. Females adopting a fixed threshold criterion use the same threshold regardless of the quality of males that are available [23]. Females adopting an adjustable threshold criterion adjust their threshold to the quality of available males [10,11,17]. Both tactics predict that females always mate with the last encountered male, whereas the number of sampled males can vary. This implies that some females accept the first male encountered and that the selected males should be of higher quality than males rejected on first visits [17].

Mate sampling has been investigated in several taxonomic groups [9,24-26]. Consistent with predictions of theoretical models, empirical studies have revealed that the female mate sampling tactic is influenced by the costs of searching [21,27-31] as well as the variation in quality among potential males [32-35]. Females should become choosier with an increase in benefits and a decrease in costs [36,37]. In contrast to Janetos’s [10] conclusions, the best-of-N tactic does not inevitably lead to the highest fitness for females. When search costs are considered, an adjustable threshold tactic (one-step decision tactic) generates higher fitness gain [11]. If search costs are very high, females should accept the first male they encounter.

Theoretical models of mate choice [10,18,22] assume that females assess males sequentially. However, especially in lekking and chorusing species of different taxonomic groups, females have opportunities to assess several males simultaneously without closely approaching them [24,38-40] and can thereby avoid costs associated with a close approach [41]. In these species, sampling is more likely to be limited by the perceptual abilities of females in distinguishing males, and females must weigh the potential for improvements in detection of male quality against the costs of reaching males [24].

Observations of anuran mate sampling suggest that both sequential [42] and simultaneous [24,43,44], assessment can occur, making them an outstanding system for studies on mate sampling tactics. Several studies have revealed that preferences of female anurans are based on male advertisement calls [40]. Males that produce energetically very costly calls are often more attractive for females [45]. Different call properties require differing energy investments and females were shown to prefer calls with more components, higher intensity, longer duration or higher rate of repetition [40]. Some properties of acoustic signals (e.g. dominant frequency of calls) can contain information about male body size [46] which often influences female mate choice decisions [40,47].

In the present study we investigated female preferences for certain male characteristics (acoustic and physical traits, territory size) as well as mate sampling tactics in the strawberry dart-poison frog (*Oophaga pumilio*). Within the last years, the ecology, reproductive and territorial behaviour, sexual selection as well as geographic, phenotypic and genetic variation in *O*. *pumilio* has received a great deal of attention [48-64]. *O*. *pumilio* provides an excellent model system for examining mate choice tactics for several reasons. These diurnal aposematically coloured frogs can easily be monitored over several months of the prolonged breeding season when females exhibit, contrary to most other anuran, very high mating rates (average interval between two matings of a female approx. 4–5 days in our monomorphic, red coloured study population [65]). Due to the lack of an amplexus males have no possibility of forcing a female to mate, thus females are free to choose among potential mates. Mating takes place in the territories of the males and after oviposition males moisten the terrestrial eggs in the leaf litter. Ten days after oviposition females start transporting their tadpoles to small water-filled leaf axils of bromeliads or bananas (only one tadpole per axil) and feed them with nutritive eggs [65-68]. *O*. *pumilio* has a lek-like mating system with long-term territoriality. Males use advertisement calls to attract females, and advertisement calls, aggressive calls and physical combat to defend their territories against intruding males. Males compete for access to mating partners and their distribution depends on the distribution of the females [49,55] while females defend feeding territories [69] and are limited in their distribution to areas with sites suited for tadpole-rearing [55]. In our study population females possess large overlapping home ranges (~36 m^2^; covering several male territories) but smaller territories (~2 m^2^) than males (~15 m^2^) [49,55,69].

In lek-like mating systems females have opportunities to compare several males sequentially and simultaneously. If female *O*. *pumilio* sample males sequentially we expect to find movements among potential mates and acceptance of mates according to the predictions of theoretical models [10,11,16,22]. However, it is possible that females sample several males simultaneously from a greater distance. Therefore we also investigated whether females preferred and chose individuals with certain characteristics (see above) regardless of whether females had visited these males before or not.

Because most data on mate choice in amphibians come from experiments, Richards-Zawacki et al. [60] emphasised the need and importance of field studies for understanding the patterns of mate choice exhibited in natural populations. However, due to the complexity of female mate choice, observations of mate sampling behaviour alone may also be insufficient and experiments are required to distinguish among different mate choice tactics [17,43,70,71]. Thus, in addition to field observations on female behaviour we used playback experiments to verify female preferences for certain male call traits. Since in a previous study male mating success of strawberry poison frogs was positively correlated with dominant frequency and call rate [51], we tested female preferences for these two call properties.

In many taxonomic groups observations on female mate sampling are restricted to a certain period before mating [e.g. [24,28,39,72,73]]. To our knowledge no information is available on the behaviour of females for the complete period between two matings. In the present study, we continuously observed individual females of the strawberry poison frog during the whole period between two successive ovipositions and simultaneously monitored the behaviour and spatial distribution of the surrounding males.

## Results

### General results

We observed the mating behaviour of 20 females (N = 5 in 2004; N = 15 in 2005). The mean intervals between two successive ovipositions were 5.8 ± 2.44 days. Ovipositions were initiated between 06:12 and 11:30 am. During courtship males led females to oviposition places (typically dry leaves) inside their territory. Males left the oviposition site 18 ± 4 min (median ± interquartile range; N = 19) min after entering the oviposition leaf. Thus they stayed a significantly shorter time at the oviposition site (Wilcoxon matched pairs test: T = 0, Z = 3.82, N = 19, p = 0.0001) than females (median ± interquartile range: 45 ± 13 min; N = 19). Clutch size varied between 3 and 8 eggs (mean: 4.89 ± 1.09 eggs (N = 20)). In only 4 different occasions we observed males leading more than one courting female successively to exactly the same oviposition place. These females deposited their clutches closely next to each other and it was not possible to distinguish between both clutches.

Hatching success was determined 7 days after oviposition because females (N = 4) revisited their clutches 7.5 ± 0.6 days after oviposition. In both years hatching success was very low. Only 20.38 ± 25.17% of the eggs per clutch (N = 19 females) survived until the seventh day after oviposition. Main reasons for egg mortality were mould, desiccation as well as predation (e.g. by ants). Two clutches did not develop.

Males stopped calling during and after oviposition of their mate. The duration of these calling interruptions was on average 32.57 ± 13.1 min (N = 14). Three males did not begin to call again after oviposition in the course of the data collection on the particular day. These males were omitted from the calculations.

### Female mate choice behaviour

#### Number of contact males

We observed the mate choice behaviour of 5 (2004) and 15 (2005) females. In the year 2005 for each female 6.4 ± 1.92 and 11.0 ± 2.62 males were available as mates in a radius of 5 and 10 m, respectively, measured from the border of their territory. However, females showed restricted mate search tactics (Table 1). Twenty-five percent of the focal females (N = 20) were in contact (approximation closer than 50 cm to a male) with only one male during the observation period (time between two successive ovipositions) and chose this male as mate. Additionally, 10% (N = 20) of females were in contact with males only during the time of mating. Twenty-five percent of females (N = 20) accepted a male with which they had no previous contact during the observation period (time between two successive ovipositions).

**Table 1:** Number of contact males

**# Contact males**	**2004**			**2005**		
	**Mean**	**S**_**D**_	**Range**	**Mean**	**S**_**D**_	**Range**
total	3.4	1.52	1–5	2.27	1.1	1–5
per day	0.78	0.28	0.33–1	1.02	0.4	0.38–1.67
at the oviposition day	1.4	0.89	1–3	1.13	0.35	1–2

Number of contacted males per focal female in the year 2004 (N = 5) and 2005 (N = 15) during the study period between two successive ovipositions. Chosen males of both ovipositions were included.

**Figure 1:**
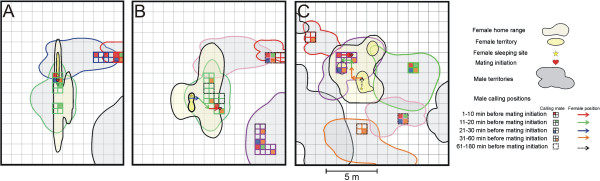
**Map of movements of three females.** Movement pattern of females relative to the calling positions of males 0–180 min before mating initiation. **A**) female mated within its territory, **B**) female left its territory for mating initiation, **C**) female with multiple territories and mating initiation outside of its territories.

On the oviposition day females were in contact with significantly more males than on other days (Friedman ANOVA: F = 7.89; N = 20, df = 2; P = 0.02). Nonetheless, on the day of oviposition 85% of females (N = 20) were in contact with only one male and chose this male as a mate. Thus, they accepted the first male they encountered (Figure 1). The other 15% of females (N = 20) were in contact with 1.33 ± 0.58 other males prior to mating on the day of oviposition and accepted the second or third contact male.

All initiated courtships resulted in oviposition except two cases when females laid unfertilised eggs without a male. In both cases courtship was interrupted by external disturbances (e.g. territorial fighting between males, biting ants, or several females trying to mate with the same male simultaneously). The females left the first male and moved fast and directly toward the next male. Also courtship with these males was disturbed by one or more of the mentioned factors. Abruptly both females showed no further courtship behaviour although the males were still calling and oviposition took place in the absence of a male in the late afternoon.

Comparing first and second ovipositions 10 of 20 focal females chose the same male again: In 93% of the cases (N = 15 females, year 2005) the male selected during the first oviposition was calling 0–60 min before initiation of the second mating.

Based on the systematic observation of males, information about 2.47 ± 1.81 (range: 0–6) additional matings of focal females (N = 14) after the regular observation period (from one oviposition to the next oviposition) was available in 2005. Thus, females were observed to choose 2 ± 0.65 (range: 1–3) different males as mates. Only 3 females oviposited repeatedly with the same male. Territories of all mates overlapped with the home ranges of females and were located in a radius of 5 m measured from the border of females’ territory.

### Female preferences

#### Distance

Eighty-two percent of all females (N = 11) spent the night before oviposition inside their territories. On the day of oviposition four of 15 females did not leave their territory for mating initiation and approached and accepted the closest calling male as mate (Figure 1). Eleven females initiated mating outside their territory. On the day of oviposition nine of these 11 females left their territory within the last 45 min (22.78 ± 9.91 min) before mating initiation and moved directly towards the (at that moment) closest calling male (N = 7 females) or towards one of the two or three closest calling males that were located at the same distance from the female (N = 2 females) and accepted this male as mate.

#### Quality of males

The average snout-vent length (SVL) of males (N = 16) in the study area was 23.82 ± 0.80 mm. Their mean weight was 1.04 ± 0.08 g. The average male condition (condition index [74-76]) amounted to −0.02 ± 0.09 g. Descriptive statistics and coefficient of variation (CV) for all call properties are given in Table 2. The ratio CVb/CVw (CV at the between-male level/CV at the within-male level) was always smaller than 2. Thus, differences in CVs between and within males were small. CVs for the number of pulses were higher and CVs for dominant frequency were lower than CVs for other properties.

**Table 2:** Male call properties

				**CV**_**w**_		**CV**_**b**_	**CV**_**b**_**/CV**_**w**_
	**N**	**Mean**	**SD**	**Mean**	**SD**		
call duration [s]	16	0.07	0.01	10.7	3.56	8.02	0.75
pulse rate [pulse/ms]	16	0.23	0.03	8.48	2.12	14.95	1.76
number of pulses	16	15.41	2.83	13.11	3.64	18.37	1.40
frequency [kHz]	16	3.97	0.14	3.28	0.93	3.53	1.07
call rate [calls/s]	16	6.46	0.39	8.18	3.08	6.06	0.74
duty cycle [s/s]	16	0.43	0.03	8.98	2.99	7.52	0.84

Descriptive statistics and coefficient of variation (within (CV_w_) and between males (CV_b_)) of call properties of male *O*. *pumilio* defending a territory in the study area.

Although females were in contact with only a few males they may be able to assess calls of males simultaneously without closely approaching them. Because focal females did not mate with a male more distant than the second closest to them (distance calculation on the day of oviposition at the moment when females left their territory; see above), we compared the qualities of the two closest males irrespective of prior contact between a female and a male. Using univariate analysis we did not find female preferences for certain call properties or physical properties (SVL, weight, body condition; Table 3).

**Table 3:** Preferences for male quality

**Parameter**	**N**	**Chosen **♂	**Rejected** ♂	**t**	**P**
call duration [s]	12	0.07 ± 0.01	0.07 ± 0.01	−0.32	0.75
number of pulses	15	14.93 ± 3.32	15.44 ± 3.31	0.32	0.76
pulse rate [pulse/ms]	15	0.22 ± 0.04	0.23 ± 0.04	0.38	0.71
frequency [kHz]	15	3.98 ± 0.16	3.96 ± 0.13	−0.31	0.76
call rate [calls/s]	15	6.44 ± 0.45	6.43 ± 0.43	−0.04	0.97
duty cycle [s/s]	15	0.43 ± 0.03	0.42 ± 0.02	−0.99	0.34
SVL [mm]	15	23.87 ± 0.99	24.16 ± 1.12	0.64	0.53
weight [g]	15	1.03 ± 0.07	1.08 ± 0.08	1.42	0.18
condition [g]	15	−0.03 ± 0.07	−0.01 ± 0.05	0.88	0.4
territory size [m^2^]	13	12.78 ± 6.99	13.23 ± 6.62	0.15	0.88
calling activity [periods/day]	10	11.92 ± 2.99	12.54 ± 2.04	0.44	0.67

Comparison between the qualities of the male chosen and rejected by each focal female using a paired *t*-test. Only the two closest calling males of each female were included.

Additionally, chosen males did not differ from the average of all other contact males (Additional file 1) or the average of all other males whose territory overlapped with the home range of the females (Additional file 2) regarding the measured traits.

Our PCA analyses yielded 10 dimensions in total, however 74% of male trait variability was representable by the first three dimensions (Table 4, Additional files 3, 4, 5). These components did not correlate with success rate, thus, mate choice by females is not significantly explainable by these factors. This is in accordance with our results of the univariate analyses (see above). We did not find differences in PCA scores between chosen and rejected males (Table 5).

**Table 4:** Principal Component Analysis of male traits

	**PCA 1**	**PCA 2**	**PCA 3**	**r**^**2**^	**p-value**
eigenvalue	3.72	2.50	1.93		
explained variance	33.86	22.77	17.57		
cumulative variance	33.86	56.63	74.20		
success rate correlation					
contact males	−0.37	−0.60	−0.71	0.56	0.12
all males	−0.13	−0.99	−0.023	0.30	0.46
contribution of traits to dimensions:					
call duration [s]	12,29	15,94	0,09		
pulse rate [pulse/ms]	14,37	9,75	0,71		
frequency [kHz]	3,18	18,48	11,55		
call rate [calls/s]	12,54	3,57	3,91		
number of pulses	21,36	1,51	0,68		
duty cycle [s/s]	0,95	31,19	1,33		
SVL [mm]	12,15	0,23	1,44		
weight [g]	5,08	3,75	32,51		
condition [g]	0,54	2,01	39,91		
territory size [m^2^]	1,53	12,99	0,48		
calling activity [periods/day]	16,02	0,55	7,39		

PCA 1–3 are the first three dimensions obtained from our analysis. Beside the typical PCA statistics, correlation score with success-rate are indicated for each dimension and with a combined r^2^ and p-value in the upper part of the table. Percental contributions of male traits to PCA dimensions are listed in the lower part of the table.

**Table 5:** Comparison of PCA scores between chosen and rejected males

	**Closest males**			**Contact males**			**All males**		
	**N**	**t**	**P**	**N**	**t**	**P**	**N**	**t**	**P**
PC 1	15	0.22	0.83	11	2.13	0.06	14	0.25	0.81
PC 2	15	−1.44	0.17	11	−0.67	0.52	14	−0.39	0.71
PC 3	15	−1.3	0.22	11	−1.12	0.29	14	−0.11	0.91

Comparison of PCA scores between the chosen and the rejected male taking into account the two closest calling males (closest males), the chosen male and the average of all other contact males (contact males), and the chosen male and the average of all other males whose territories overlapped with the territory of the female (all males) using the paired *t*-test.

According to our power analyses for the t-tests (with n = 15, p < 0.05, and power = 0.8, paired design), the sample size is good for our parameters with high effect size (territory size and calling activity) and marginally adequate for medium effect sizes (number of pulses and pulse rate). The effect size is low for the remaining parameters (frequency, call duration, call rate, condition, SVL, weight).

### Playback experiments

**Figure 2:**
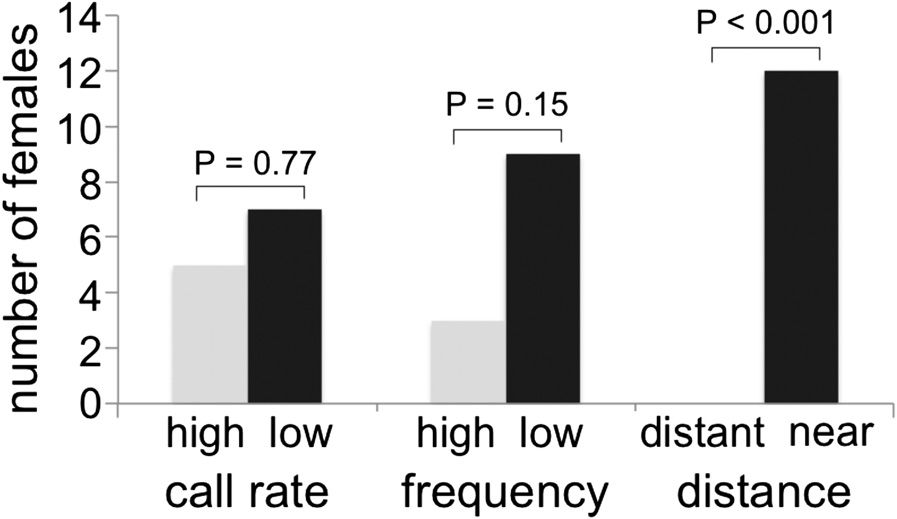
**Playback experiments.** Number of females choosing one of the two alternatives (high vs. low call rate, high vs. low frequency, distant vs. near speaker). A two-sided binomial test was used to test for female preferences.

Thirty-six of 45 females tested in playback experiments responded by approaching a speaker. Females showed no preference for the higher or lower call rate or higher or lower dominant frequency (Figure 2). However, they showed a clear preference for the closest speaker (Figure 2).

During each experiment there was no difference between females choosing one speaker and females choosing the other speaker with regard to their body condition, SVL, weight, time and air temperature (*t*-test or U-test; p > 0.05).

## Discussion

### Preferences

Our results suggest that the distance to potential mates is the crucial criterion for mate choice in female strawberry poison frogs in our study population. The tested acoustic and physical traits, and territory size did not seem to influence female mate choice decision.

In many taxa female preferences are based on male acoustic properties [reviewed by [77]]. Pröhl [51] found that in *O*. *pumilio* males, mating success is positively correlated with calling activity and call rate and negatively with dominant frequency in some study years. However, female preferences for call traits are not the only possible explanation for this observation. Instead, males with certain characteristics (call properties, fighting abilities) could have the ability to defend preferred areas with high female density [49,55] and thus gain a mating advantage. In many anuran species [78-80] including *O*. *pumilio*[49] males reduce their dominant call frequency during social interactions with other males. Possibly, frequency is involved in the assessment of fighting ability between rival males [80,81]. Since male strawberry poison frogs that defend territories with high female density also have a higher mating success [55], the correlation between certain call properties of males and their mating success [51] could be due to intrasexual interactions.

In most anurans, male vocalizations are the primary signal modality. However, many studies suggest that different modalities are involved in information transfer and sexual selection in anurans [82-87] and other taxonomic groups [reviewed by [88]]. Recent experiments with polymorphic and monomorphic Panamanian populations of *O*. *pumilio* revealed that females prefer males of their own phenotype to males with other colours [59,60,89-91]. Beside colouration, other parameters such as brightness or conspicuousness may also be important cues for female mate choice [58]. However, the extreme colour variability in this species seems to be restricted to the islands of the Bocas del Toro archipelago and the adjacent mainland of Panama [92,93]. Whether brightness, conspicuousness and colouration influences female mate choice decisions in the monomorphic red or reddish orange populations of the remaining distribution range in Costa Rica and Nicaragua remains to be addressed in future studies.

Meuche et al. [49] showed that males possess smaller territories in areas with high female density and strong intrasexual competition for these places. In the present study accepted males tend to occupy smaller territories than their neighbours. This is consistent with a situation where males compete for places with high female density because the best competitors would be closest to more females than males that defend territories with low female density.

Females might prefer other parameters such as resources defended by males [9,77,94]. The quality of oviposition sites inside the territory of each male could influence female preferences if the quality of the defended area has an impact on hatching success [95,96]. Additionally, male parental care can affect hatching success of clutches and female preferences [97]. With one exception (*Hyla rosenbergi*[98]) there are no indications that female anurans prefer better fathers as mates. In *O*. *pumilio* overall hatching success is very low [this study, [99]] and probably depending on many ecological factors that cannot be controlled by males (e.g. predation, drought). Oviposition sites (mainly rolled-up leaves of bananas, *Heliconia* and caoutchouc trees) are very abundant and do not seem to be a limited resource [100]. Furthermore, during our study no female rejected a male after visiting the oviposition site. Therefore, we assume that female mate decisions are not influenced by male parental care and the quality of oviposition sites. Additionally, there is no evidence that mate choice in *O*. *pumilio* is based on tadpole rearing sites since 1) males do not control the access to larval habitats and 2) females usually transport tadpoles to water bodies outside the territory of the fathers [55].

We are aware that sample sizes are low for a part of our statistical analyses and their non-significance should be considered with caution. However, as some of the parameters with low effect-size are connected to the high or medium effect-size parameters (I. Meuche, unpublished data), we expect them to be similar regarding their effect on mating success. Strong positive or negative effects should be identifiable at least in some of the parameters, if present. We thus consider low effect-size parameters also as not significant and assume this effect to be consistent even when studying larger sample sizes.

#### Mate sampling tactic

Our data suggest that in our study population, female strawberry poison frogs use a mate sampling tactic that could be defined as “accept the closest calling male”. The probability that a female mated with a male was not influenced by the analysed characteristics (Table 3) and 85% of all focal females (N = 20) accepted the first male they encountered on the day of oviposition. Non-choosy females or the acceptance of the closest male as mate sampling tactic has also been observed in fishes [101,102] and some anuran species with a lek-like mating system [24,103,104]. For example in the dendrobatid frog *Allobates femoralis* spatial and genetic pattern of reproductive success suggest that females are likely to mate with any spatially proximate territorial male and only territory possession determines if males are considered as mating partner [105,106]. Additionally, recent studies suggest that females of *Hyla gratiosa* use a simultaneous sampling tactic [24,43]. Observations showed that females moved to a location where they were able to compare several males simultaneously, selecting and approaching the preferred male directly. Such a tactic could also be applied by females of *O*. *pumilio*. However in that case we would expect females to choose their mates according to certain characteristics. Our data did not reveal any female preference for male traits that might be possible indicators of fitness (Tables 3, 4, 5).

The choice of the closest caller as a mate was not the result of preliminary movements towards the preferred male. Most females spent the night and morning before oviposition inside their territories before approaching the closest caller.

Experience from previous sampling bouts early in the mating season has been shown to reduce mate sampling in later time periods especially in species were male display sites are stable [107,108]. In *O*. *pumilio* both males [109] and females (I. Meuche pers. obs.) are defending their territories for several months or years. Although, long-term experience could explain restricted mate sampling found in females of *O*. *pumilio*, it is not consistent with the clear preference for the closest caller. Additionally, it is unlikely that the choice of the closest calling male is simply the result of establishing a territory near the preferred males because female distribution does not depend on male distribution [55] and seems to be associated with feeding sites [69]. However, further research is necessary to determine the influence of long-term experience on mate sampling in *O*. *pumilio*.

Selective phonotactic movements toward the closest male could be a cost-reducing strategy in terms of search time and competition for mates. Computer simulations have shown that the number of sampled males decreases as sampling costs increase [110]. Especially time constraints seem to affect the optimal sampling tactic in different taxonomic groups [9,29,111-114]. Even in mating systems where females can simultaneously evaluate a large number of mates, the time-related costs would force them to assess only a small number of males [115] for a relatively short time [44]. In *O*. *pumilio*, time constraints seemed to increase the risk of failure to mate. Females that were not able to obtain a mate within a certain period of time laid unfertilised eggs in the absence of a male.

Additionally, indirect competition between females for access to males can cause time constraints if the probability of finding an available male decreases with increasing competition [103,116]. The intensity of this indirect competition depends on the sex ratio of adults. There is an increased risk of failure to mate when the sex ratio is more biased towards the choosing sex [9]. For example in fishes [117-120] and birds [27] it has been shown that choosiness is reduced to minimise the risk of not reproducing when the sex ratio is more biased towards the choosing sex. Female strawberry poison frogs are only able to localise males, which call. We found that males did not call for an average of 31 min during and after oviposition of their mate and thus were not localisable from greater distances by other females. Additionally, many males called only if females were in sight. This observation, the strongly female biased sex ratio [109], and the fact that most matings concentrate on days with optimal climatic conditions [121], reduced the probability of finding an available male over greater distances in our study population. In contrast to our results, Pröhl & Hödl [65] reported mate sampling and courtship interruptions by females from a population with an 1:1 sex ratio. We conclude that the mate sampling tactic might be a flexible tactic depending on the actual sex ratio, thereby offering more opportunities for females to be choosy in male biased populations. In contrast, mate sampling and choosiness decrease in more female biased populations [119]. The female biased sex ratio in our study area seems to rely on the much higher abundance of potential tadpole rearing sites (i.e. female reproductive resources) in comparison to areas where the sex ratio is equal [55,109]. Since female home ranges largely overlap [69] more females might fit into an area of a certain size. However, the number of males in the same area might be restricted due to their pronounced territoriality and bioacoustic spacing.

Energetic limitations or predation can also influence optimal sampling tactics. Sampling several males can reduce energy reserves that could be allocated otherwise into the offspring [122]. In some studies energetic constraints influenced mate choice [28,123,124]. For example, female sticklebacks are highly selective between dull and bright males when sampling costs are low. However, females reduced their selectivity when they had expended energy swimming against a current [123]. No information is available on energetic costs of female mate sampling in the strawberry poison frog; however, it can be assumed that energetic expenditures increase when females are covering greater distances. As females of *O*. *pumilio* defend their territory against other females [69] and the sex ratio is strongly female biased, intensive mate sampling could lead to increasing energetic expenditures [see [11]], stress and risk of losing the territory to another female.

In different taxonomic groups it has been shown that energetic constraints imposed by parasites are reducing female selectivity [[125-127], but see [128]]. In *O*. *pumilio* ecto- and endoparasites have been found [129-131], and parasite infections seem to vary among individuals [131] and populations (S. Hagemann, pers. comm.). In our study population we never found ectoparasites in the skin of any of the females or males. Additionally, the load of intestinal parasites load was very low in both sexes [I. Meuche unpublished data, [131]] in comparison to other anuran species from the same geographic region [132]. Because condition-dependent characteristics of males are not associated with parasite load [131] we assume that parasites did not impose energetic constraints on the females in our study population.

Predation risk has also been shown to influence female choosiness in other taxa [133,134]. In contrast to other anurans [104,135,136] predation risk for the aposematically coloured populations of strawberry poison frogs seems to be low [137]. However, populations differ in their colouration, toxicity, and local predator communities [63,93,138-141]. Recent studies showed that the red colour morph posseses a more effective warning signal and a lower predation rate than other colour morphs [137,138]. These findings are supported by the fact that in our study area the mortality rate of all territorial males in the course of 12 months was 0%. Therefore, we assume that predation risk and its influence on female mate choice was very low in our red coloured study population. However, further studies are necessary to determine whether the mate sampling tactic used by female strawberry poison frogs is a flexible tactic depending on colouration, availability of defensive alkaloids, and/or predation pressure.

In addition to the costs of intensive mate sampling, benefits also affect the intensity and precision of a preference [33,142]. When the benefits of intensive mate sampling do not exceed the costs of being choosy, females should be less selective and sample fewer males [11,36]. Theoretical models show that selectivity increases as variation among potential mates increases because benefits of mating with high quality males increases [11]. If the variance in male quality is low, and the costs of comparing males are high [104], selection should favour females mating with the first male encountered [47]. In accordance with Pröhl [51], the variation in bioacoustic traits among males of *O*. *pumilio* was low in our study population. Additionally, males defending territories with high female density over several years [65] are expected to be high quality males. In many populations, very low-quality males are eliminated by male-male competition, so all individuals owning a territory are acceptable [47,105]. Consequently, females get high-quality mates even if they accept the closest calling male [see [135]].

## Conclusions

High egg mortality as well as the risk of losing the whole clutch by laying unfertilised eggs and the probably low benefits of intensive mate sampling support our assumption that acceptance of the closest calling male represents an optimal mate sampling tactic in female strawberry poison frogs in the investigated population. However, given the large variation in traits (e.g. sex ratio, colouration, toxicity, predation) among different populations of *O*. *pumilio*, further studies in a natural situation as well as experimental set-up are needed to evaluate the potential within-species differences in sampling tactics.

## Methods

### Study site

The present study was conducted at the Hitoy Cerere Biological Reserve on the Caribbean side of Costa Rica (9°40^′^ N, 83°05^′^ W) between August 2004 and May 2006. We established two study areas (located at 100 m above sea level) near the Hitoy Cerere river. The study areas were chosen with regard to high frog density (> 2 males per 100 m^2^[109]) and accessibility for the researchers. The density was confirmed by capturing and re-capturing frogs at the beginning of the study.

The vegetation consisted mostly of bananas (*Musa spp*., Musaceae) and *Heliconia spp*. (Heliconiaceae) as well as *Carludovica rotundifolia* (Cyclanthaceae) and caoutchouc trees (*Castilla elastica*). The two areas measured 2400 m^2^ (2004) and 505 m^2^ (2005) and were divided by nylon strings into 1 m^2^ quadrats for the recording of spatial distribution of all frogs.

### Field observations

The study was carried out in accordance with the legal and ethical standards of Costa Rica and was approved by the corresponding government authorities (MINAE/SINAC permit No. 137-2004-OFAU, 038-2005-OFAU, 004 ASP AC/AC). In the following we describe the behavioural observations of males and females. In general, we minimised the impact of our presence on animal behaviour by moving slowly. As a result, no individuals jumped away or seemed to hide when we approached.

### Females

From September to December 2004 as well as from April to August 2005, we observed the mate choice behaviour of 20 females for an average of 70 h per female. We successively monitored the behaviour of each focal female from one oviposition to the next oviposition (approximately 5 days) using “focal animal sampling” [143]. The position of the female in the grid system was recorded every 5 min during daylight hours (05:00 am – 06:00 pm). It was not necessary to observe individual frogs during the night because they do not leave their sleeping sites in the leaf litter until dawn (I. Meuche, pers. obs.). Individuals were easily distinguishable by their natural marks such as black spots.

Behavioural observations were made every minute using “one–zero-sampling” [143]. We recorded contacts with males (yes/no), the identity of these males, courtship (yes/no) and mating behaviour (yes/no). In *O*. *pumilio* certain male traits (e.g. weight, condition) are not correlated with male advertisement call properties [51] and, therefore, cannot be assessed by females over large distances. As the dense secondary forest floor creates a visual barrier, it is unlikely that females can assess these properties from a distance of more than 50 cm. Thus, contact with males was defined as an approach closer than 50 cm to a male (or vice versa) [49]. Courtship was an interaction in which a female showed a directional movement toward a calling male and followed this male at least for a short distance [65]. Tactile, visual, and acoustic stimuli were also possible [66]. A mating was defined as a courtship followed by an oviposition event. Because both sexes were visually undetectable after entering the oviposition leaf, the start of an oviposition was defined when both sexes entered the leaf. The oviposition was completed when the leaf was abandoned after egg laying. After each oviposition the number of eggs was determined and the clutches were inspected daily determining the rates and probable causes for mortality.

Data collection was stopped when the focal female transported tadpoles during the observation period (time between two successive ovipositions) as tadpole rearing females do not continue to mate [65].

To minimise the effect of our presence on female behaviour during data collection, focal females were not captured until the end of each observation period. We measured their weight to the nearest 0.01 g with a Voltcraft PS-250 scale and SVL to the nearest 0.1 mm with a manual calliper. Each focal female was marked by toe-clipping for individual identification – a conventional marking method [144] that does not affect the behaviour of *O*. *pumilio*[109]. We used toe-clipping in this study only after a careful consideration of welfare implications. We only marked two toes or fingers per frog and only one per limb. Furthermore, the first finger was never marked to avoid impacting their climbing abilities. Additionally, each female was photographed for re-identification via its individual patterns of black spots and short lines on the red dorsal ground colour. However, due to changes of individual patterns over time [145], photos alone were insufficient for unequivocal re-identification in our study population.

### Males

At the beginning of the behavioural observations, male frogs (N = 16) were captured for measuring their weight and SVL. Individuals were recognised by distinctive natural marks such as black spots (using photographs) and their toe-clipping pattern. Males were observed over a period of 65 days (from April 2005 to August 2005) by 1–2 other persons during the same time when female behaviour was observed. All observers were trained prior to the observation period ensuring thereby high consistency in data recording.

As calling activity was most intense from 07:00 to 10:00 a.m. and mating as well as oviposition only occurs in the morning [54], male behaviour was studied from 06:00 to 10:00 a.m. Males were successively located at five minute intervals (“scan sampling” [143]) and their position in the grid system was recorded. Observation time per male was approximately 15 seconds during each interval. The sampling order of males was maintained during each five minute interval. We recorded calling activity (yes/no) using “one–zero sampling“ [143]. Furthermore, we recorded every mating activity (yes/no) with our focal females to obtain information about additional matings of focal females after the regular observation period (from one oviposition to the next oviposition).

Vocalisations of males were recorded during the morning using a Sony Professional Walkman (WM DC6) and a Sennheiser Directional Microphone (MZA 14 P48) placed approximately 50 cm from the males. We recorded at least six advertisement call sequences for each male on different days. Subsequently, air temperature was measured using a digital thermometer (Voltcraft DT-8820 Environment Meter) with an accuracy of 0.1°C.

### Data analyses

#### Sound analyses

We analysed 190 advertisement call sequences of 17 males (mean: 11.2 sequences/male) using the Digital Sound Analysing System Avisoft-SASLab Pro (Fa. Specht, Berlin, Germany) at a sampling rate of 44100 Hz. Power spectrograms (hamming window, FFT-length: 256) were used to measure dominant frequency. Temporal properties were measured using an oscillogram. A call sequence is a series of single calls produced in succession during several seconds or minutes. Between call sequences males pause for irregular time intervals. A call is defined as a complete sequence of pulses separated from the next call by an intercall interval [see [51]]. Ten consecutive calls of each call sequence were analysed, quantifying the following call properties: call duration (s), dominant frequency (kHz), number of pulses per call and pulse rate (pulses/ms). Additionally, we determined the call rate (calls/s) by counting the number of calls during three periods of two seconds. The mean of the above mentioned call properties was calculated for every call sequence. The duty cycle was determined as an indicator of the percentage of time an acoustic signal was produced within a call sequence. It was calculated as the call rate multiplied by average call duration per call sequence [see [146,147]]. To avoid variability caused by different thermal conditions, all temperature-dependent call parameters [49] were adjusted to a temperature of 25.6°C (average temperature during call recordings) using standard regression methods. For every male and each call parameter the average value of the call sequences was used. Additionally, we determined a coefficient of variation (CV) of the call properties at the within-male level (CV_w_) and between-male level (CV_B_), and calculated the ratio CV_B_/CV_w_.

### Territories and home ranges

For each male in the study area as well as each focal female, we calculated the position and size of territories as well as home ranges, respectively. The position data from the grid system were transformed in x- and y-coordinates, in which each coordinate corresponds to the centre of the grid cell. Home range and territory sizes were calculated with the adaptive-kernel method [148] using the software ArcView GIS 3.3 (Fa. Environmental Systems Research Institute (ESRI)) as well as the supplementary programs Spatial Analyst 2.0a (Fa. ESRI), Cad2Shape 3.0, and Home Range Extension [149].

Using the kernel method we calculated the density function for 95% of the observation points allowing us to determine the interpolated home ranges (focal females) or territories (males) [49,69]. Additionally, we calculated the density function for 50% of the observation points of focal females and defined this area as the females’ territory [69].

### Body condition

Since individual conditions can influence female mate choice [39] a condition index [74-76] was calculated. The condition of each individual was determined by regression between the initial body mass of all males and females and their SVL (linear regression in males: F = 21.5; R = 0.61; R^2^ = 0.37; T = 4.6; N = 38; P < 0.001; y = −0.795351992 + 0.0778729712*x), (linear regression in females: R = 0.68; R^2^ = 0.46; N = 38; P < 0.001; y = −0.4712 + 0.0664*x). The condition of an individual was defined as the deviation of its body mass from the body mass predicted by the above regression (residuals).

### Mate choice

We quantified the total and daily number of different contact males to determine mate search pattern of focal females in 2004 and 2005. Additionally, we compared the number of contact males on different days (oviposition day, one day before oviposition, and ≥ 2 days before oviposition) using a Friedman-ANOVA. We averaged the number of contact males for the days 2–11 before oviposition because depending on the female the time-interval between two successive ovipositions was 2–11 days.

In 2005 we also localised the sleeping places of females, allowing us to eliminate the possibility that females shift their sleeping places toward preferred males in the night before mating. Additionally, we determined whether females initiated mating inside or outside their territory. For females that initiated mating outside their territory we determined the moment when females left their territory and approached a calling male on the oviposition day. Then, we calculated the distance between the focal females and all calling males in the study area based on their actual location (position coordinates corresponded to the center of the grid cell) at that moment. For females that mated inside their territory, we calculated the distance between the current position (center of the grid cell) of the focal females and all calling males at the moment when females started approaching the male. We did this to find out whether the females mated with the closest male. It is generally unknown when the females of our study species make their decision, which in turn might depend on the moment of ovulation. However, at the day of oviposition most females stayed within their territory before directly approaching and choosing a mate (see results). Therefore, we assume that females make their decision before leaving their territories or prior to approaching a male.

With regard to male qualities we determined the condition, SVL, body weight, territory size as well as male call properties (call duration, dominant frequency, the number of pulses per call, pulse rate, call rate, duty cycle), and calling activity (the number of five minute periods of calling activity).

The focal females did not mate with a calling male more distant than the second closest to them (distance calculation at the moment when females left their territory; see above). Consequently, we compared the above mentioned qualities of these two closest males, regardless of whether females were in contact with them before or not, using a paired *t*-test. However, females could assess males over a longer period than just the day of oviposition. Therefore, a paired *t*-test demonstrated whether there was a difference between the qualities of the chosen male and the average qualities of all other contact males or the average qualities of all other males whose territories overlapped with the home range of each focal female. Females that were in contact only with the chosen male or whose home range overlapped with the territory of only one male were omitted from these analyses. A power analysis was conducted to determine if the sample size was adequate to show statistically significant differences.

Furthermore, in multivariate analyses we compared the success-rate (number of successes/total number of comparisons) of (1) all contact males and (2) all males within the home range of the female according to female choices for male traits. For this, we computed a principal component analysis (PCA) for male traits prior to correlation with success-rate because (1) many of these traits were shown to correlate with one another [51], (2) different studies have highlighted the importance of multiple traits in female mate choice decisions [46,150,151] and (3) PCA reduces the probability of inflated correlations due to excessive number of traits. Our PCA was computed with R (R Development Core Team, 2012). The parameters “territory size“, “clutch survival rate “and “calling activity“, included missing values for some males. We accounted for that by imputing missing entries using an iterative PCA algorithm on bootstrapped PCA models as described in Josse et al. [152] and implemented in the R package “missMDA” [153]. Related diagnostics plots can be found in the Additional files 3, 4, 5. Success-rate was correlated with the first three dimensions of the PCA by using the “vegan” package [154]. P-values were based on 999 permutations. The R-code for this analysis is available upon request from the authors. Finally, by using a paired *t*-test we compared the PCA scores between (1) the two closest calling males, (2) the chosen male and the average of all other contact males, and (3) the chosen male and the average of all other within the home range of the female. A power analysis was conducted using the R package “pwr” [155]. If not stated otherwise, means ± S_D_ (standard deviation) are given.

### Playback experiments

#### Synthetic calls

**Figure 3:**
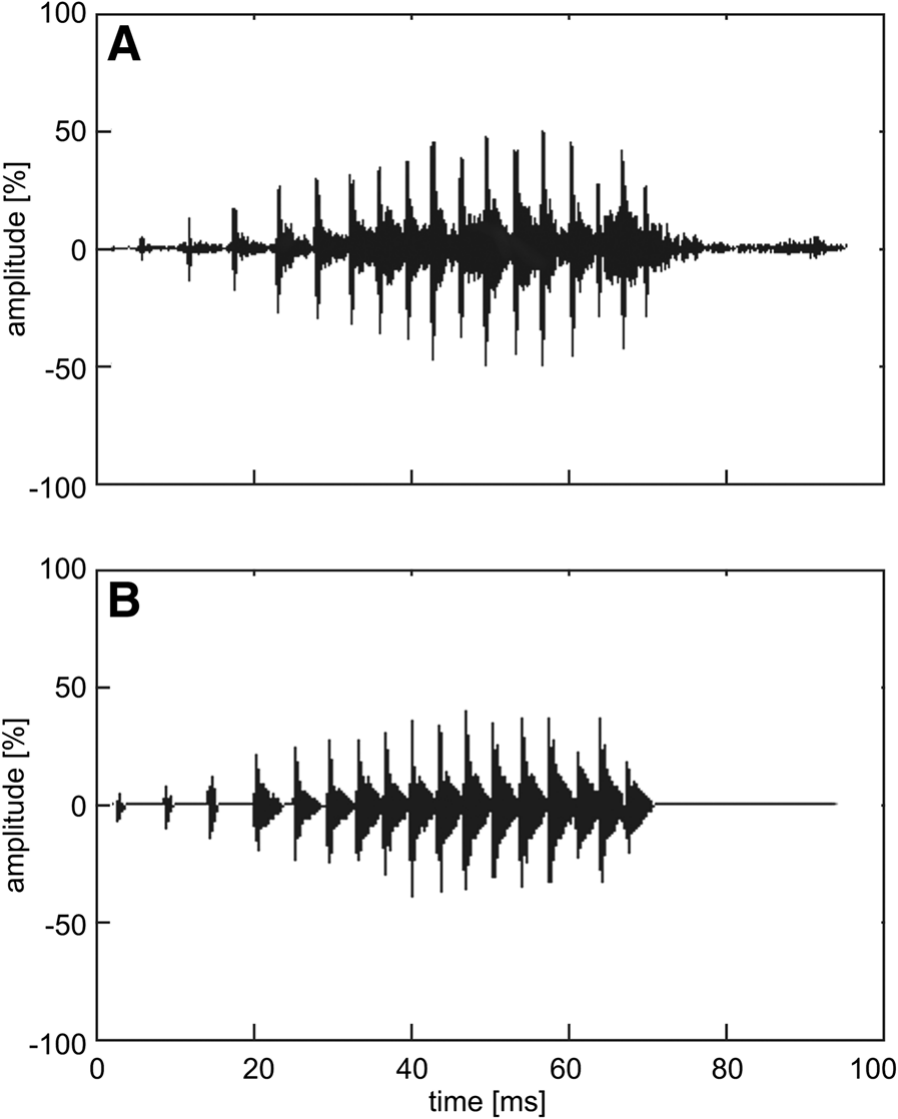
**Oscillogram. A**. Oscillogram of a typical call depicting the average characteristics found in the population; **B**. Oscillogram of the synthetic call used in the playback experiments.

Acoustic stimuli employed in playback experiments were synthesised using the software Avisoft-SASLab Pro (Fa. Sprecht, Berlin, Germany) and were based on a typical call according to average characteristics found in the same population by Pröhl [51] (Figure 3). The call sequences were recorded non-stop onto high quality Sony UX-S Chrome Class Premium audio tapes using the Software Goldwave (Fa. Goldwave Inc.).

#### Experimental procedure

From November 2005 to May 2006 between 08:00 am and 01:00 pm hours phonotactic responses of females were tested in two-choice discrimination experiments under natural conditions in the home range of each female. Because preliminary playback tests demonstrated that non-courting females did not show positive phonotactic movements, we only tested receptive i.e. courting females. Females were considered to be courting when they approached and followed a calling male [156]. Before each test we captured the present male as well as all other calling males within a radius of 5 m. To avoid disturbances we left the female untouched at the site. Some females were tested more than once but never for the same stimulus pair to avoid pseudo replication.

After the experiments females were captured, identified or marked and photographed, measured (SVL), and weighed. Additionally, air temperature as well as the time and duration of each test were recorded.

#### Call rate experiment

**Figure 4:**
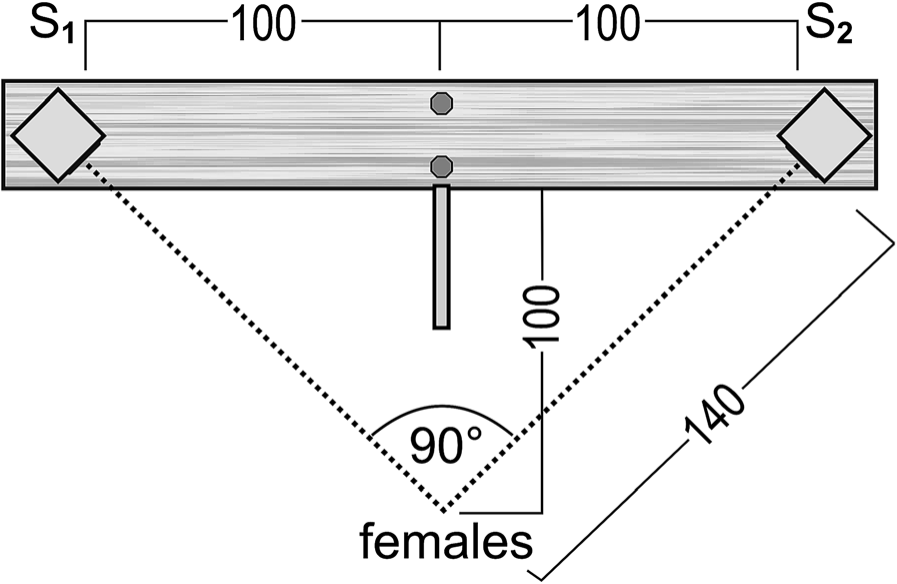
**Set-up of the frequency and call rate experiments.** Schematic representation of the playback experiments (call rate and frequency). All measurements are in cm. Two speakers (S) were located in a triangle of 45° at the ends of a plank. A 50 cm long stick (dark-grey line) was used to obtain the correct distance of 1 m between the female and the plank. Two nails (dark-grey circles) acted as a makeshift to obtain the triangle of 90° between the female and the plank. The triangle was formed when the head of both nails were in line with the female.

To ensure equal distances between both speakers as well as between the speaker and the female in each test, we located two water-resistant broadband loudspeakers (VISATON FRS 10 WP) in a triangle of 45°. The loudspeakers were placed on a 14 cm × 212 cm long plank with a distance of two metres between each other and were removable at both ends (Figure 4). The distance between both speakers was comparable to the natural distances (1–4 m) between neighbouring territorial males [52,65,157]. Each speaker was connected to a Sony Professional Walkman WM-D6C, an amplifier (Roadstar AM-311) and a 12V battery. Two nails and a 50 cm long stick were placed in the middle of the plank in a 90° triangle to allow adjustments of the set up.

Before each test we measured the sound pressure level (SPL) of the two speakers with the RadioShack sound level meter (measurement error: ± 2db at 114 db) and set the volume to 70 db at a distance of 50 cm [52]. The set-up had been adjusted prior to the experimental start. The 50 cm long stick was used to obtain the correct distance of 1m between the center of the plank and the female. Both nails acted as a makeshift to ensure the correct angle of 90° between the female and both loudspeakers. Subsequently, synthetic calls were presented simultaneously from both speakers at different call rates (5 calls/sec vs. 8 calls/sec (mean of the population ± 2SD)). In accordance with other studies on dendrobatid frogs [e.g. [158]] we scored a positive phonotactic response when females approached a speaker within a 20 cm radius or less. Between each experiment, the side used for one stimulus was switched. A two-sided binominal test was used to determine significant preferences.

#### Dominant frequency experiment

The same procedure was used for the recording of dominant frequency as in the call rate experiment. However, the SPL was adjusted to 67 db at a distance of 140 cm (female start point). We presented females with two synthetic calls that differed in dominant frequency (3.6 kHz vs. 4.4 kHz (mean of the population ± 2SD); natural range of the study population: 3.2–4.5 kHz). All other call properties corresponded to the average values found in the natural population [51].

#### Distance experiment

**Figure 5:**
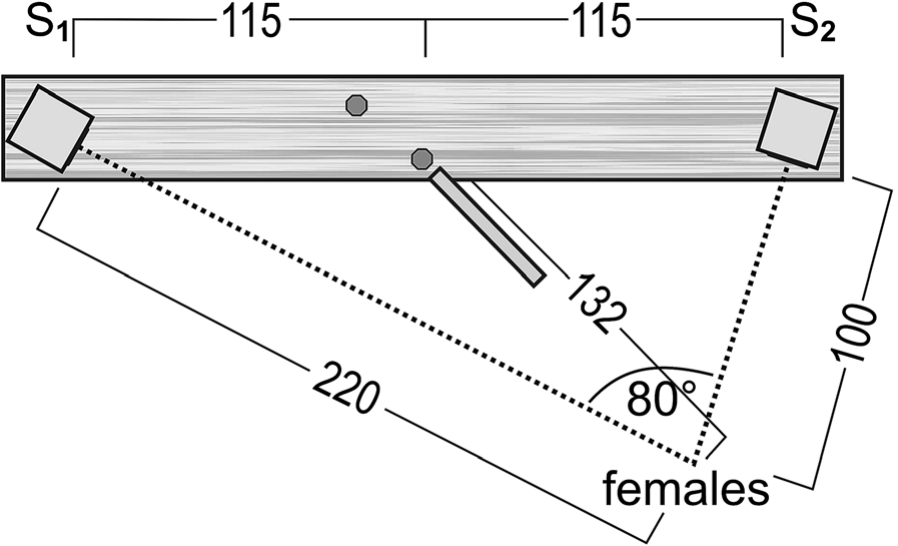
**Set-up of the distance experiments.** Schematic representation of the playback experiments (distance). All measurements are in cm. Two speakers (S) were located at the ends of a plank. A 66 cm long stick (dark-grey line) was used to obtain the correct distance of 1.32 cm between the female and the plank. Two nails (dark-grey circles) were used to obtain a triangle of 80° between the female and both speakers. The triangle was formed when the head of both nails were in line with the female.

This experiment was conducted to test whether female mate choice is affected by the distance to potential mates. One speaker was closer (1 m) to the female than the other speaker (2.2 m) (Figure 5). The distance between both speakers was 2.3 m. The female and the speakers formed an angle of 80°. Two nails and a 66 cm long stick were placed in the middle of the plank for adjusting the set-up. The SPL of each speaker was adjusted to 70 db at a distance of 50 cm. Both speakers broadcasted identical calls with standard characteristics and a frequency of 4.4 kHz. The SPL of the closer speaker was 55 dB and the SPL of the distant speaker was 63 dB at the starting point of the female.

## Competing interests

The authors declare that they have no competing interests.

## Authors’ contributions

IM, OB, AK, KEL and HP participated in design of the study and/or statistical analysis. IM and AK performed the data collection. IM wrote the manuscript. OB, AK, KEL and HP helped to draft the manuscript. All authors read and approved the final manuscript.

## Supplementary Material

Additional file 1 **Comparing the chosen male with all other contact males.** Comparison between the qualities of the chosen male and the average quality of all other contact males (rejected) of each focal female using a paired *t*-test. Click here for file

Additional file 2 **Comparing the chosen male with all males within the female`s home.** Comparison between the qualities of the chosen male and the average quality of all other males (rejected) whose territories overlapped with focal the home range of the focal female using a paired *t*-test. P-values significant after Bonferroni adjustment: p < a = 0.05/11 = 0.0045. Click here for file

Additional file 3 **First two dimensions of PCA analysis.** PCA with imputed and bootstrapped missing values for territory size, clutch survival rate, and calling activity. Male traits are indicated as vectors. Variables without missing values only show a single coloured point at the tip of the arrows. Variables with missing values (territory size = pink, clutch survival = orange, and calling activity = turquoise) show a cloud of values that reflects the variability with which missing values can be predicted. Click here for file

Additional file 4 **Location of the males (m) according to the first two dimensions of the PCA.** Ranges are according to multiple imputations for missing values of territory size, clutch survival rate and calling activity. Click here for file

Additional file 5 **Eigenvalues of all calculated PCA ordination dimensions.** According to the “elbow“-criterion, the first three dimensions were considered as most relevant and used for correlation with success rate. Click here for file
