# Supplementary material for: Only distance matters – non-choosy females in a poison frog population
Source: Front Zool. 2013 May 20;10:29. doi: 10.1186/1742-9994-10-29 (PMC3665588; doi:10.1186/1742-9994-10-29)
Supplement: Additional file 1 — Comparing the chosen male with all other contact males. Comparison between the qualities of the chosen male and the average quality of all other contact males (rejected) of each focal female using a paired t-test. [file 1742-9994-10-29-S1.doc]

**Additional file 1**

| parameter | N | chosen ♂ | rejected ♂ | t | P |  |
| --- | --- | --- | --- | --- | --- | --- |
| call duration [s] | 11 | 0.07 ± 0.01 | 0.07 ± 0.01 | -0.31 | 0.76 |  |
| number of pulses | 11 | 14.43 ± 2.7 | 16.5 ± 3.45 | 1.18 | 0.27 |  |
| pulse rate [pulse/ms] | 11 | 0.21 ± 0.03 | 0.24 ± 0.03 | 1.88 | 0.09 |  |
| frequency [kHz] | 11 | 3.93 ± 0.15 | 3.98 ± 0.12 | 0.97 | 0.35 |  |
| call rate [calls/s] | 11 | 6.37 ± 0.37 | 6.42 ± 0.48 | 0.28 | 0.79 |  |
| duty cycle [s/s] | 11 | 0.43 ± 0.04 | 0.42 ± 0.02 | -0.81 | 0.44 |  |
| SVL [mm] | 11 | 23.71 ± 1.06 | 24.11 ± 0.84 | 0.85 | 0.41 |  |
| weight [g] | 11 | 1.04 ± 0.08 | 1.03 ± 0.07 | -0.06 | 0.95 |  |
| condition [g] | 11 | -0.02 ± 0.07 | -0.05 ± 0.05 | -1.26 | 0.23 |  |
| territory size [m²] | 11 | 10.68 ± 5.7 | 15.81 ± 4.41 | 2.12 | 0.06 |  |
| calling activity [periods/day] | 7 | 11.66 ± 3.61 | 12.43 ± 1.91 | 0.4 | 0.71 |  |
|  |  |  |  |  |  |  |
