# Supplementary material for: Only distance matters – non-choosy females in a poison frog population
Source: Front Zool. 2013 May 20;10:29. doi: 10.1186/1742-9994-10-29 (PMC3665588; doi:10.1186/1742-9994-10-29)
Supplement: Additional file 2 — Comparing the chosen male with all males within the female`s home. Comparison between the qualities of the chosen male and the average quality of all other males (rejected) whose territories overlapped with focal the home range of the focal female using a paired t-test. P-values significant after Bonferroni adjustment: p < a = 0.05/11 = 0.0045. [file 1742-9994-10-29-S2.doc]

**Additional file 2**

| parameter | N | chosen ♂ | rejected ♂ | t | P |  |
| --- | --- | --- | --- | --- | --- | --- |
| call duration [s] | 14 | 0.07 ± 0.01 | 0.07 ± 0.004 | -0.73 | 0.48 |  |
| number of pulses | 14 | 15.14 ± 3.35 | 16.01 ± 2.43 | 0.62 | 0.54 |  |
| pulse rate [pulse/ms] | 14 | 0.22 ± 0.04 | 0.24 ± 0.03 | 0.9 | 0.39 |  |
| frequency [kHz] | 14 | 3.97 ± 0.16 | 3.98 ± 0.1 | 0.13 | 0.9 |  |
| call rate [calls/s] | 14 | 6.38 ± 0.41 | 6.38 ± 0.29 | -0.06 | 0.95 |  |
| duty cycle [s/s] | 14 | 0.43 ± 0.03 | 0.41 ± 0.02 | -1.51 | 0.15 |  |
| SVL [mm] | 14 | 23.79 ± 0.99 | 23.96 ± 0.61 | 0.46 | 0.65 |  |
| weight [g] | 14 | 1.03 ± 0.07 | 1.04 ± 0.04 | 0.31 | 0.76 |  |
| condition [g] | 14 | -0.03 ± 0.07 | -0.04 ± 0.04 | -0.51 | 0.62 |  |
| territory size [m²] | 13 | 12.48 ± 6.8 | 17.97 ± 3.79 | 2.55 | 0.03 |  |
| calling activity [periods/day] | 10 | 11.92 ± 2.99 | 11.93 ± 1.38 | 0.01 | 0.99 |  |
|  |  |  |  |  |  |  |
