# Supplementary figures and images for: Only distance matters – non-choosy females in a poison frog population
Source: Front Zool. 2013 May 20;10:29. doi: 10.1186/1742-9994-10-29 (PMC3665588; doi:10.1186/1742-9994-10-29)

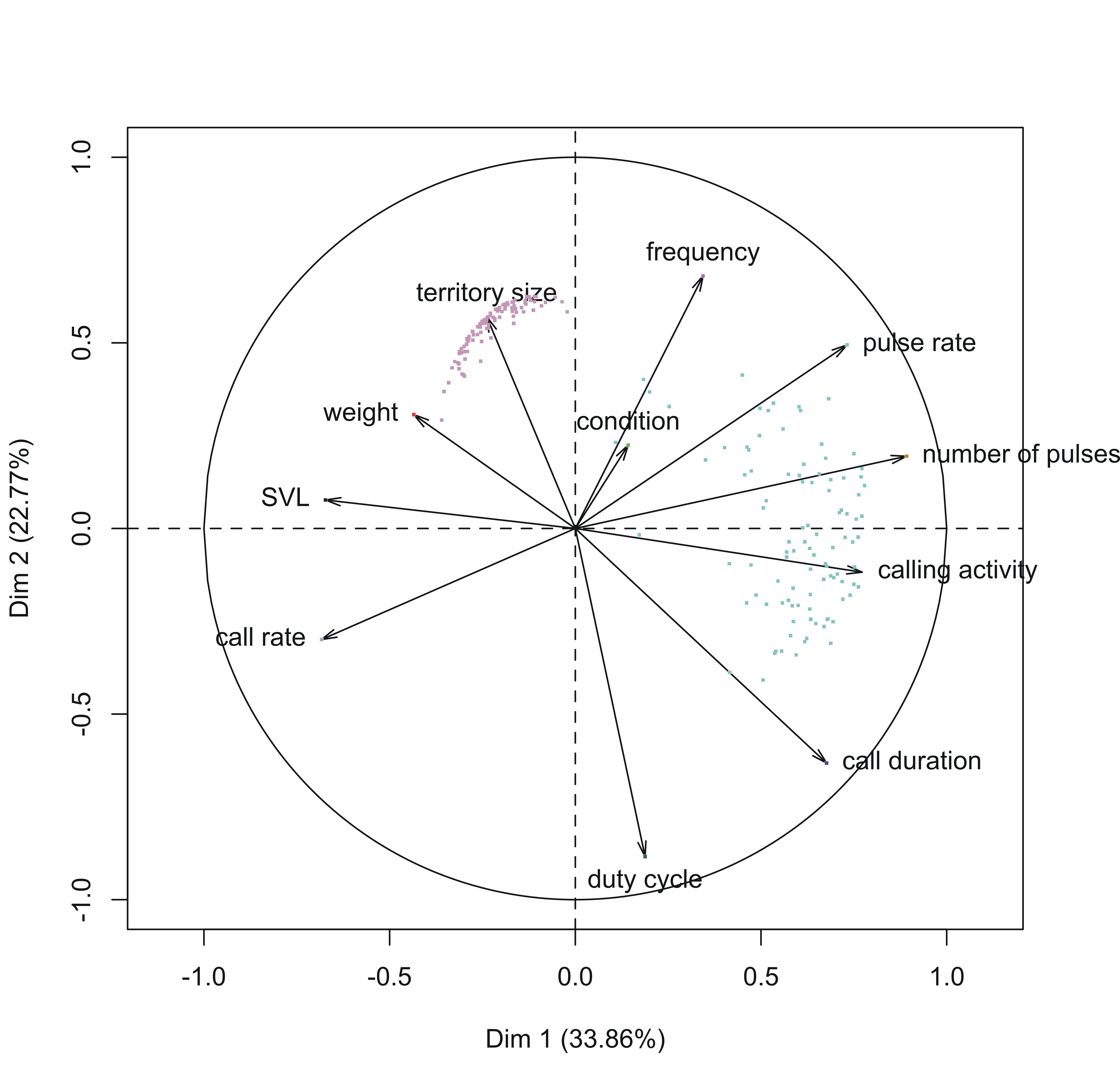

Supplement: Additional file 3 — First two dimensions of PCA analysis. PCA with imputed and bootstrapped missing values for territory size, clutch survival rate, and calling activity. Male traits are indicated as vectors. Variables without missing values only show a single coloured point at the tip of the arrows. Variables with missing values (territory size = pink, clutch survival = orange, and calling activity = turquoise) show a cloud of values that reflects the variability with which missing values can be predicted. [file 1742-9994-10-29-S3.png]

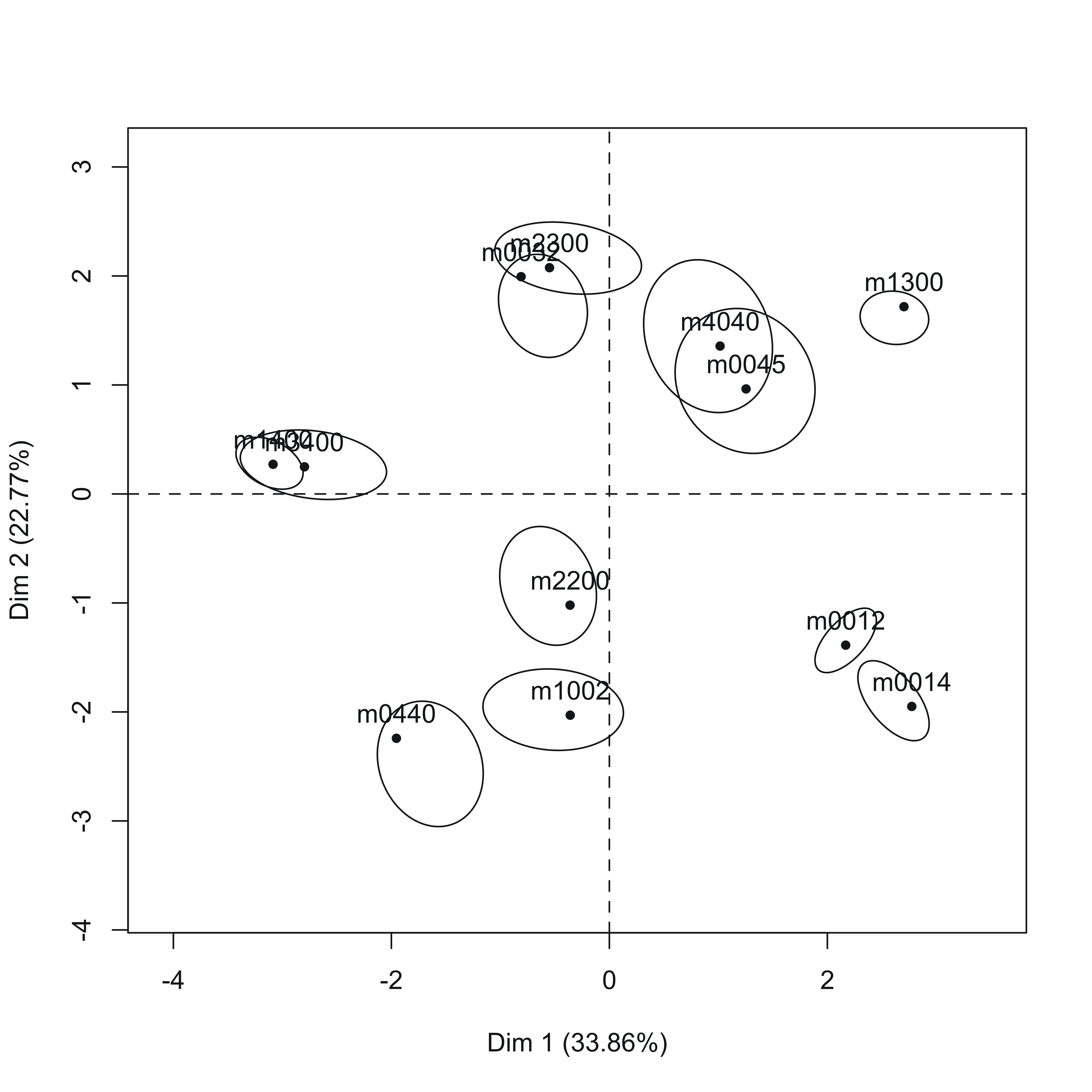

Supplement: Additional file 4 — Location of the males (m) according to the first two dimensions of the PCA. Ranges are according to multiple imputations for missing values of territory size, clutch survival rate and calling activity. [file 1742-9994-10-29-S4.png]

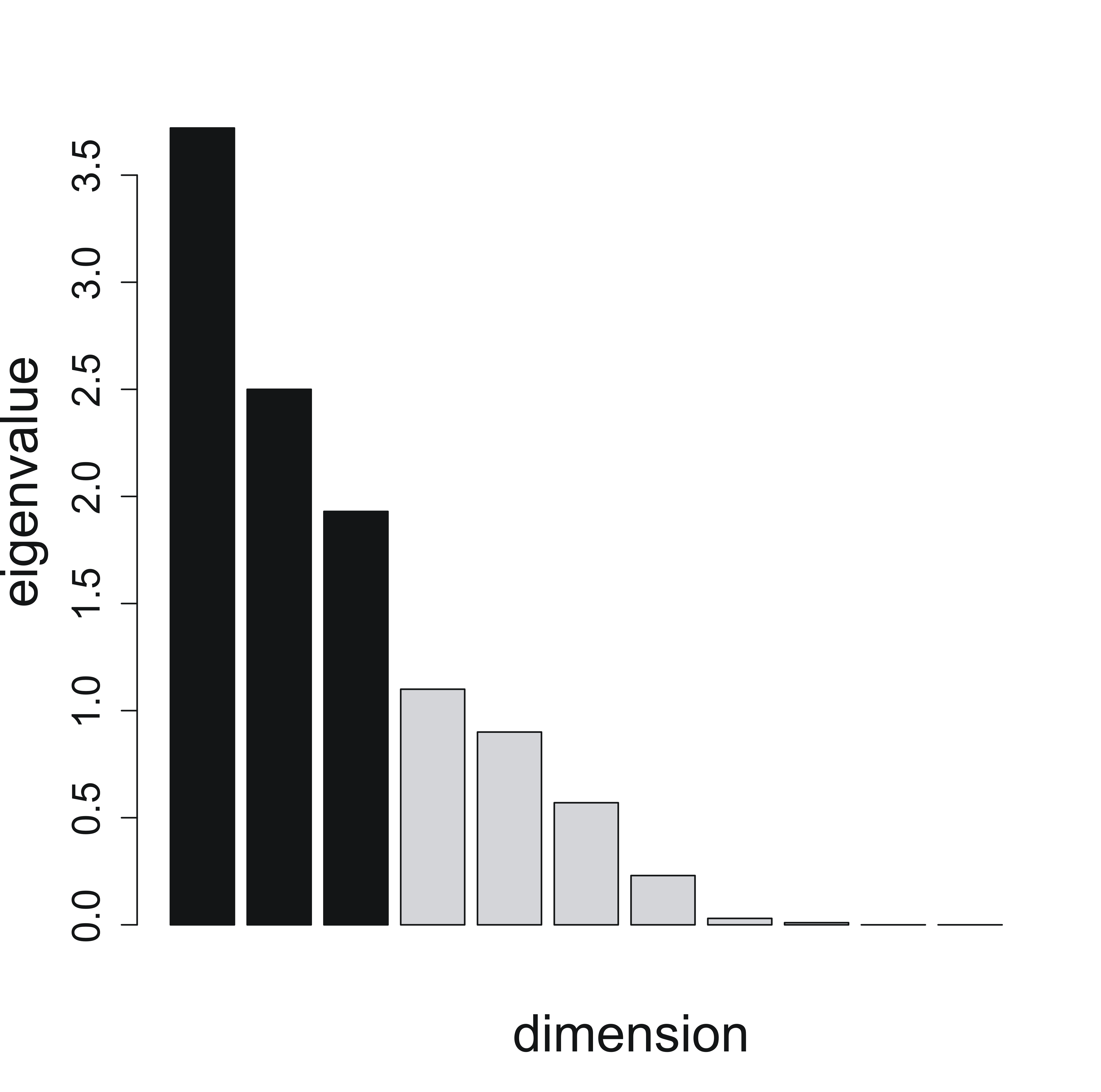

Supplement: Additional file 5 — Eigenvalues of all calculated PCA ordination dimensions. According to the “elbow“-criterion, the first three dimensions were considered as most relevant and used for correlation with success rate. [file 1742-9994-10-29-S5.png]
